# Glucosamine Improves Non-Alcoholic Fatty Liver Disease Induced by High-Fat and High-Sugar Diet through Regulating Intestinal Barrier Function, Liver Inflammation, and Lipid Metabolism

**DOI:** 10.3390/molecules28196918

**Published:** 2023-10-03

**Authors:** Feng Li, Zhengyan Zhang, Yan Bai, Qishi Che, Hua Cao, Jiao Guo, Zhengquan Su

**Affiliations:** 1 Guangdong Engineering Research Center of Natural Products and New Drugs, Guangdong Pharmaceutical University, Guangzhou 510006, China; 2 Guangdong Metabolic Disease Research Center of Integrated Chinese and Western Medicine, Guangdong TCM Key Laboratory for Metabolic Diseases, Guangdong Pharmaceutical University, Guangzhou 510006, China; 3 School of Public Health, Guangdong Pharmaceutical University, Guangzhou 510310, China; 4 Guangzhou Rainhome Pharm & Tech Co., Ltd., Science City, Guangzhou 510663, China; 5 School of Chemistry and Chemical Engineering, Guangdong Pharmaceutical University, Zhongshan 528458, China; caohua@gdpu.edu.cn

**Keywords:** non-alcoholic fatty liver disease, glucosamine, inflammation, liver lipid metabolism, intestinal barrier

## Abstract

Non-alcoholic fatty liver disease (NAFLD) is a liver disease syndrome. The prevalence of NAFLD has continued to increase globally, and NAFLD has become a worldwide public health problem. Glucosamine (GLC) is an amino monosaccharide derivative of glucose. GLC has been proven to not only be effective in anti-inflammation applications, but also to modulate the gut microbiota effectively. Therefore, in this study, the therapeutic effect of GLC in the NAFLD context and the mechanisms underlying these effects were explored. Specifically, an NAFLD model was established by feeding mice a high-fat and high-sugar diet (HFHSD), and the HFHSD-fed NAFLD mice were treated with GLC. First, we investigated the effect of treating NAFLD mice with GLC by analyzing serum- and liver-related indicator levels. We found that GLC attenuated insulin resistance and inflammation, increased antioxidant function, and attenuated serum and liver lipid metabolism in the mice. Then, we investigated the mechanism underlying liver lipid metabolism, inflammation, and intestinal barrier function in these mice. We found that GLC can improve liver lipid metabolism and relieve insulin resistance and oxidative stress levels. In addition, GLC treatment increased intestinal barrier function, reduced LPS translocation, and reduced liver inflammation by inhibiting the activation of the LPS/TLR4/NF-κB pathway, thereby effectively ameliorating liver lesions in NAFLD mice.

## 1. Introduction

It is estimated that more than 25% of adults worldwide are affected by non-alcoholic fatty liver disease (NAFLD) [1]. The development of NAFLD in our country and even in the world has shown an astonishing growth rate within a short period [2]. The pathogenesis of NAFLD is still not fully understood. The “multiple hits” theory [3] holds that multiple injuries act together to induce NAFLD in genetically susceptible subjects, including specific genetic and epigenetic factors, insulin resistance (IR), inflammation, oxidative stress, endoplasmic reticulum stress, environmental factors, TM6SF2 [4,5], gut microbes and their metabolites [6].

The liver is the central station of lipid metabolism, and the accumulation of liver lipids caused by enhanced lipid synthesis and reduced lipid catabolism can be accelerated through various stress reactions and IR, leading to the degeneration and deterioration of the liver, ultimately causing NAFLD [7,8]. Inflammation plays an important role in the occurrence and development of NAFLD as it leads to liver steatosis, which is the first NAFLD stage. Then, the accumulation of fatty acids in the liver and IR activate the production and release of proinflammatory cytokines in the liver, leading to the occurrence and development of chronic liver inflammation. NF-κB is one of the main regulatory factors of liver inflammation and is involved in the progression of NAFLD-related liver inflammation [9]. The gut–liver axis plays an important role in the occurrence and progression of NAFLD [10], specifically including gut barrier function, endotoxin translocation, changes in bacterial composition, and the influence of bacterial metabolites [11]. Disruption of the gut barrier can lead to lipopolysaccharide (LPS) translocation, systemic endotoxemia, and chronic liver inflammation. It has been proven that the plasma endotoxin level in NAFLD patients is significantly increased, indicating that the endotoxin level is related to the severity of liver steatosis [12]. To date, no specific drug has been approved for use in treating NAFLD patients [13]. The treatment for patients with NAFLD remains focused on lifestyle interventions [14].

Glucosamine (GLC) is an amino monosaccharide derivative of digested glucose and is an important component in the production of glycosaminoglycans, glycosylated proteins, and lipids in the cartilage matrix and synovial fluid, which can improve osteoarthritis [15]. GLC can reduce the activation of NF-κB and the production of reactive oxygen species (ROS) in macrophages, decrease the expression of interleukin (IL)-1β, IL-6, and TNF-α, as well as inhibit the NLRP3 inflammasome assembly and IL-1β precursor expression in the body [16]. In the human tract, only 10–12% of glucosamine is absorbed [17,18]. More than 50% of glucosamine is metabolized by gut microbiota [18]. Some studies suggest that oral administration of glucosamine sulfate causes changes in the abundance of gut microbiota [19]. Notably, GLC regulates the composition and function of the intestinal microbiota in mice, specifically inhibiting inflammatory responses in the colon and adipose tissue. Therefore, an increasing number of studies have shown that glycosaminoglycans such as glucosamine and chondroitin can exert a positive influence on the gut [19]. Therefore, we wanted to explore the role of GLC in the intestine. Based on the excellent anti-inflammatory and intestinal regulatory effects of GLC, we explore the therapeutic effect of GLC on NAFLD from the perspective of inflammation, lipid metabolism, and intestinal barrier.

In this study, we used a high-fat and high-sugar diet (HFHSD) to establish an NAFLD model with mice, and the NAFLD mice were treated with GLC. This article reports the preliminarily exploration of the therapeutic effect of GLC on NAFLD by measuring the levels of inflammation, lipid metabolism, antioxidant, and other factors in serum. Then, further research was conducted by performing H&E staining, quantitative real-time PCRs, Western blotting, and immunohistochemistry to further clarify the role of GLC in improving inflammation, lipid metabolism, and intestinal barrier function in NAFLD mice. This article provides clues for the development of new treatments based on the effect of GLC. As a natural sugar, GLC exhibits a high-safety profile and induces few toxic side effects, providing a novel basis for developing safe and reliable drugs for the treatment of NAFLD.

## 2. Results

### 2.1. Changes in Food Intake, Body Weight, Serum Glucose, and Insulin Levels

**Figure 1:**
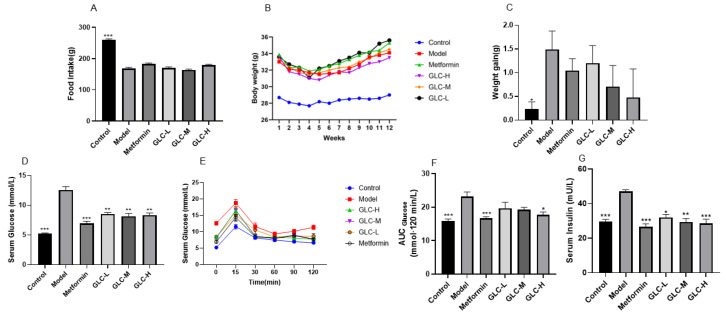
Changes in food intake level, body weight, serum glucose, and insulin levels. (**A**) The total food intake level, (**B**) weekly weight change, (**C**) weight gain, (**D**) fasting blood glucose level, (**E**) serum glucose curve, (**F**) area under the glucose curve, and (**G**) serum insulin level. The data are presented as the mean ± SEM (n = 8). * *p* < 0.05, ** *p* < 0.01, *** *p* < 0.001 compared to the model group.

During 12 weeks of oral administration of GLC, compared with that of the control group, the average food intake level of the other groups consisting of mice fed a HFHSD was lower. This may have been due to the high-calorie content of the HFHSD diet, causing a decrease in the appetite of the mice (Figure 1A). There was no evident discrepancy in the food intake level of each administration group compared with that of the model group, indicating that GLC did not have an appetite-suppressing effect on the mice. During 12 weeks of oral administration of GLC, compared with that of the control group fed an ordinary feed, the body weight of the group fed with a HFHSD was increased (Figure 1B,C). GLC administration reduced the body weight gain in the HFHSD-fed NAFLD mice to a certain extent. After oral administration of GLC for 12 weeks, the oral glucose tolerance of the mice was analyzed, and it was found that the HFHSD induced a prominent increase in serum insulin and blood glucose levels in NAFLD mice, while GLC and metformin significantly attenuated hyperinsulinemia and hyperglycemia in the NAFLD mice (Figure 1D–G). Thus, GLC administration can attenuate IR in the NAFLD mice.

### 2.2. GLC Can Improve Serum Lipid Metabolism in NAFLD Mice

**Figure 2:**
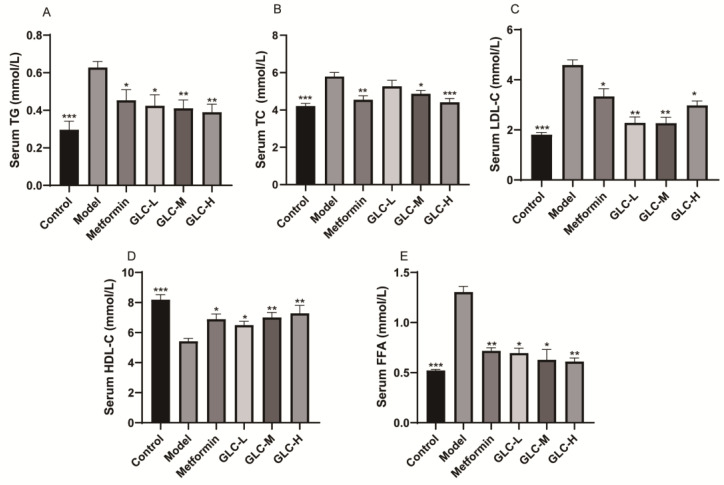
GLC can improve serum lipid metabolism in HFHSD-fed mice. (**A**) Serum TG level, (**B**) serum TC level, (**C**) serum LDL-C level, (**D**) serum HDL-C level, and (**E**) serum FFA level. The data are presented as the mean ± SEM (n = 8). * *p* < 0.05, ** *p* < 0.01, *** *p* < 0.001 compared to the model group.

We measured the levels of triglycerides (TG) (Figure 2A), total cholesterol (TC) (Figure 2B), low-density lipoprotein cholesterol (LDL-C) (Figure 2C), high-density lipoprotein cholesterol (HDL-C) (Figure 2D), and free fatty acids (FFA) (Figure 2E) in serum. The results indicate that the LDL-C, TG, TC, and FFA levels in the serum of the NAFLD mice were markedly increased, while the HDL-C level was sensibly decreased. Each dose of GLC significantly reduced the levels of serum TG, LDL-C, and FFA in mice. Both high and medium doses of GLC can reduce the serum TC level in mice. In addition, metformin reduced serum TG, TC, LDL-C, and FFA levels in mice, while GLC-H and metformin increased serum HDL-C levels in mice. These results show that GLC exerts a certain attenuating effect on abnormal blood lipid metabolism in NAFLD mice.

### 2.3. GLC Improves Serum Transaminase Levels as well as Inflammation and Oxidative Function in NAFLD Mice

**Figure 3:**
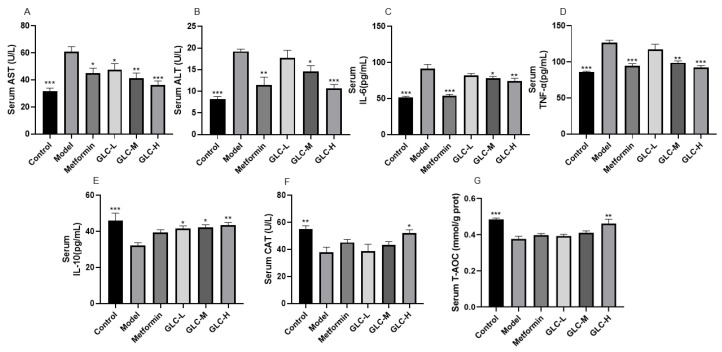
GLC improves serum transaminase levels as well as inflammation and oxidative function in NAFLD mice. (**A**) Serum AST level, (**B**) serum ALT level, (**C**) serum IL-6 level, (**D**) serum TNF-α level, (**E**) serum IL-10 level, (**F**) serum CAT level, and (**G**) serum T-AOC in each group of mice after the treatment. The data are presented as the mean ± SEM (n = 8). * *p* < 0.05, ** *p* < 0.01, *** *p* < 0.001 compared to the model group.

Transaminases are the primary metabolism enzymes in the liver. After the liver cells are damaged, the functional integrity of the cell membrane is diminished, and when the membrane is permeable, enzymes are released into the blood, leading to an evident increase in serum aspartate aminotransferase (AST) and alanine aminotransferase (ALT) levels; therefore, serum ALT and AST levels are key indicators of the degree of liver damage [20]. By measuring the serum AST and ALT levels in NAFLD mice (Figure 3A,B), we found that compared with the control group, the model group mice developed a certain degree of liver damage, which was manifested by clearly increased serum AST and ALT levels. However, GLC administration and metformin treatment markedly reduced the serum AST and ALT levels in NAFLD mice, indicating that GLC alleviated liver damage in NAFLD mice.

Inflammation can cause a hepatocyte stress response and induce lipid accumulation, which is a prominent pathological factor in the progression of NAFLD. We observed that compared with that in the control group, the serum interleukin 10 (IL-10) level in the model mouse group was moderately reduced, while the tumor necrosis factor-α (TNF-α) and interleukin 6 (IL-6) levels were moderately increased, indicating that the HFHSD-fed NAFLD mice developed systemic inflammation. After administration, mice in the GLC-H and GLC-M groups had significantly reduced serum levels of TNF-α and IL-6. Metformin can significantly reduce the serum levels of TNF-α and IL-6. Compared with that in the model group, the serum IL-10 level in the metformin-treated group was elevated and all doses of GLC moderately increased the level of IL-10 in the serum of the mice in the treatment group (Figure 3C–E). These results suggest that GLC reduces the level of proinflammatory factors in NAFLD mice, increases the release rate of anti-inflammatory factors, and effectively alleviates the systemic inflammatory response.

The results from the assessment of serum antioxidant function showed that GLC-H and metformin improved the serum catalase (CAT) level and total antioxidant capacity (T-AOC) levels of NAFLD mice to a certain extent, indicating that GLC can increase the serum antioxidant capacity of NAFLD mice (Figure 3F,G).

GLC treatment improved the levels of the aforementioned indicators, indicating that GLC attenuated liver damage in NAFLD mice, slowed the systemic inflammatory response in these mice, and increased the antioxidant capacity in the mice.

### 2.4. GLC Can Improve Liver Lipid Accumulation and Oxidative Function in NAFLD Mice

**Figure 4:**
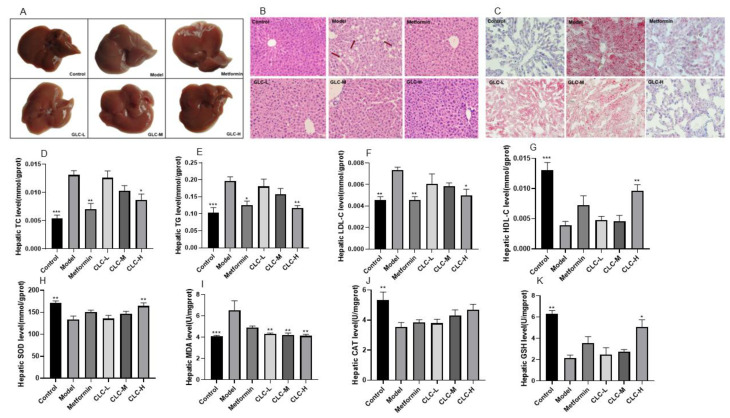
GLC improves liver lipid accumulation and oxidative function in NAFLD mice. (**A**) The overall morphology of the liver, (**B**) H&E stained tissue, (**C**) oil red O stained tissue, (**D**) liver TC level, (**E**) liver TG level, (**F**) liver LDL-C level, (**G**) liver HDL-C level, (**H**) liver SOD level, (**I**) liver MDA level, (**J**) liver CAT level, and (**K**) liver GSH level. The data are presented as the mean ± SEM (n = 6). * *p* < 0.05, ** *p* < 0.01, *** *p* < 0.001 compared to the model group.

We then examined the livers of NAFLD mice. First, we observed fresh mouse livers (Figure 4A). The morphology of the livers in the control group mice was normal, with a dark red, smooth, and glossy surface. In the model group, the color of the mouse livers was dull, with a significant greasy sensation visible on the surface, and in severe cases, there were significant fat spots on the surface. After metformin and GLC administration, especially in the GLC-H group, the liver morphology was most similar to that in the control group. Then, the mouse livers were sliced, and the sliced sections were stained. The H&E staining results showed (Figure 4B) that the hepatocytes in the livers of the control group mice were regularly and tightly arranged, while the livers of the model group mice showed many fat vacuoles and a loose arrangement, indicating severe steatosis. After GLC and metformin administration, the number of fat vacuoles in liver tissue slices of the mice was significantly reduced. Oil red O staining results showed (Figure 4C) that many lipid droplets were stained red in the model group. After GLC and metformin administration, the number of red lipid droplets in the mouse livers in each group decreased significantly, and the lipid droplets were smaller. Administration of GLC significantly reduces lipid accumulation in the liver and attenuates liver steatosis.

Furthermore, we measured the levels of the lipid metabolism indexes TG, TC, LDL-C, and HDL-C in the mouse liver (Figure 4D–G). Compared with those in the control group, the levels of LDL-C, TC, and TG in the livers of the mice in the model group were significantly increased, while the level of HDL-C was significantly decreased. After administration of GLC-H, the levels of TG, TC, LDL-C, and HDL-C in the livers of the mice in each group were significantly improved. Metformin can significantly reduce serum TG, TC, and LDL-C levels, and compared with that in the model group, the HDL-C level in the metformin treatment group was elevated. The experimental results showed that administration of GLC moderately reduced the lipid content of the livers of the mice and attenuated liver steatosis in the NAFLD mice. Additionally, we found that the levels of the liver antioxidant function indicators GSH (glutathione), CAT, and SOD (superoxide dismutase) in the model group were notably reduced, while the malondialdehyde (MDA) level was markedly increased (Figure 4H–K), indicating that the liver of the NAFLD mice was damaged by lipid peroxidation and that the liver antioxidant function was impaired. GLC and metformin administration can increase the antioxidant capacity of the NAFLD mice and exert a significant inhibitory effect on lipid peroxidation in the NAFLD mice.

### 2.5. The Effect of GLC on Liver Lipid Metabolism at the mRNA Expression Level in NAFLD Mice

**Figure 5:**
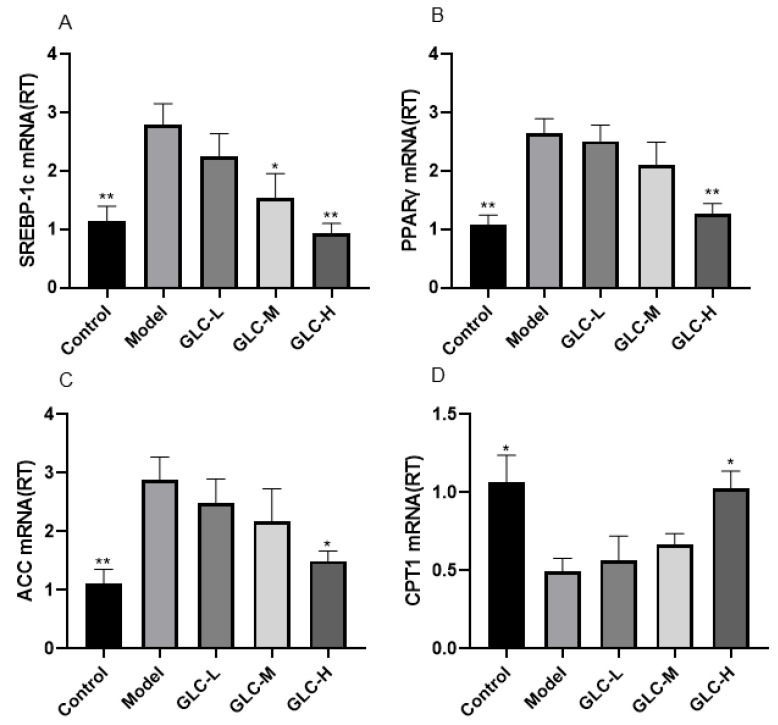
The effect of GLC on liver lipid metabolism. After GLC treatment, the expression of (**A**) SREBP-1, (**B**) PPARγ, and (**C**) ACC decreased and the expression of (**D**) CPT1 increased. The data are presented as the mean ± SEM (n = 6). * *p* < 0.05, ** *p* < 0.01 compared to the model group.

Hepatic de novo lipogenesis (DNL) is the process of endogenous synthesis of lipids from dietary sources or stored energy pools, and increased hepatic DNL is one of the clear abnormal manifestations of NAFLD [21]. Carnitine palmitoyl transferase 1 (CPT1) is the master regulator of fatty acid β-oxidation and promotes fatty acid β-oxidation in liver mitochondria [22]. A relative quantitative analysis of the mRNA expression of the liver lipid de novo synthesis genes sterol regulatory element-binding protein-1c (SREBP-1c), peroxisome proliferator-activated receptor-γ (PPARγ), acetyl-CoA carboxylase (ACC), and the liver fatty acid β-oxidation gene CPT1 was carried out by quantitative real-time PCR (Figure 5). The results showed that the expression of the SREBP-1, PPARγ, and ACC genes in the livers of model group mice was moderately upregulated, and the expression of the CPT1 gene was moderately downregulated. After GLC treatment, the change in the expression of each gene was significantly improved, indicating that GLC administration can significantly improve liver lipid metabolism disorder in the mice. GLC administration improves hepatic lipid metabolism in NAFLD mice by inhibiting hepatic DNL and accelerating hepatic fatty acid β-oxidation.

### 2.6. GLC Ameliorates Liver Inflammation in NAFLD Mice through the LPS/TLR4/NF-κB Signaling Pathway

NAFLD is a proinflammatory disease, and inflammation is critical to the occurrence and progression of NASH. In fact, steatosis alone does not adversely affect NAFLD prognosis, but inflammation and the main consequence of inflammation, namely, fibrosis are key determinants of long-term prognosis for patients with the disease [23].

**Figure 6:**
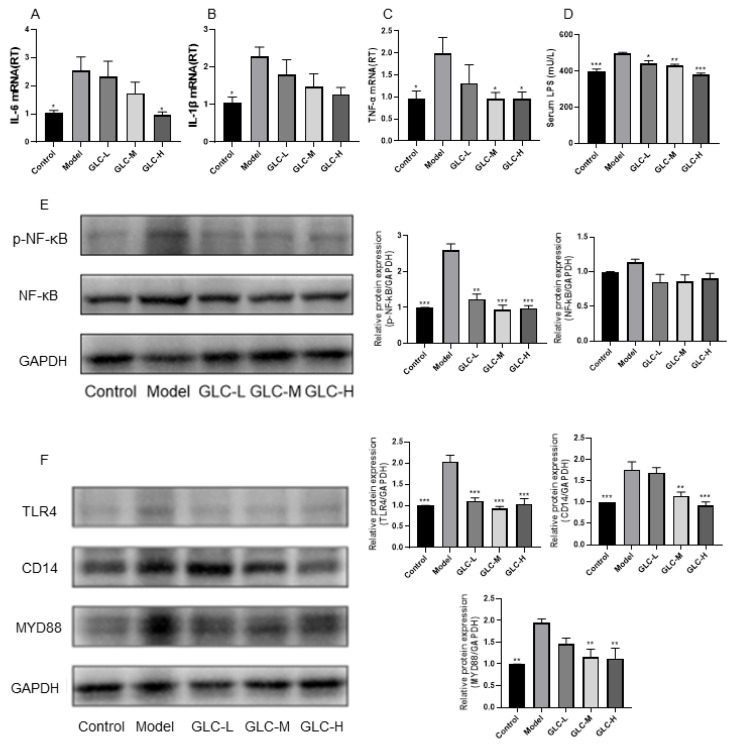
The effect of GLC on inflammation. (**A**) IL-6, (**B**) IL-1β, (**C**) TNF-α gene expression levels (n = 6), (**D**) serum LPS level, (**E**) the expression of the p-NF-κB and NF-κB proteins, (**F**) TLR4, CD14, and MYD88 protein levels (n = 4). The data are presented as the mean ± SEM. * *p* < 0.05, ** *p* < 0.01, *** *p* < 0.001 compared to the model group.

First, we performed a quantitative analysis of the relative mRNA expression of the inflammatory factors IL-6, TNF-α, and IL-1β in the liver. The RT-PCR results are shown in Figure 6A–C and indicate that the expression levels of the proinflammatory factors IL-6, TNF-α, and IL-1β in the livers of the mice in the model group were clearly increased. However, after GLC treatment, the expression of the aforementioned inflammatory factors was decreased, indicating that GLC administration may reduce liver inflammation by regulating the balance between proinflammatory and anti-inflammatory effects.

NF-κB, an important transcription factor regulating inflammation, is essential for NAFLD liver inflammation [9]. Therefore, we quantitatively analyzed the expression of hepatic NF-κB and phosphorylated NF-κB (p-NF-κB) by Western blotting assays (Figure 6E). The results indicated that the expression of p-NF-κB in the livers of the mice in the model group was moderately increased, and GLC administration moderately inhibited the activation of NF-κB. In addition, compared with that in the control group, the expression of NF-κB in the liver of the NAFLD mice increased to a certain extent, and the expression of NF-κB decreased after GLC administration, but the difference was not significant. Then, we further studied the proteins upstream and downstream of NF-κB. Specifically, we quantitatively analyzed the protein expression of TLR4, MYD88, and CD14 in the liver (Figure 6F). The protein expression levels of TLR4, MYD88, and CD14 in the livers of the model group mice were moderately increased, and the protein expression levels of TLR4, MYD88, and CD14 were markedly decreased after GLC treatment, indicating that GLC administration may improve liver inflammation by regulating the TLR4/NF-κB pathway.

Excessive LPS can induce a strong inflammatory response through the TLR4/NF-κB inflammatory pathway, cause liver injury, and accelerate the development of NAFLD [24,25]. Therefore, in this study, the serum LPS level in the mice was measured (Figure 6D). The assay results indicated that the serum LPS level of mice in the model group was moderately increased, while after GLC treatment, the serum LPS level of the mice was sensibly decreased, indicating that GLC administration effectively decreased the serum LPS level in the NAFLD mice.

From the aforementioned results, we found that GLC can attenuate the inflammatory process in the liver of HFHSD-fed NAFLD mice by LPS/TLR4/NF-κB inflammatory pathway activation and regulating the expression of related inflammatory factors in the pathway.

### 2.7. GLC Improves Gut Barrier Function Composition in NAFLD Mice

**Figure 7:**
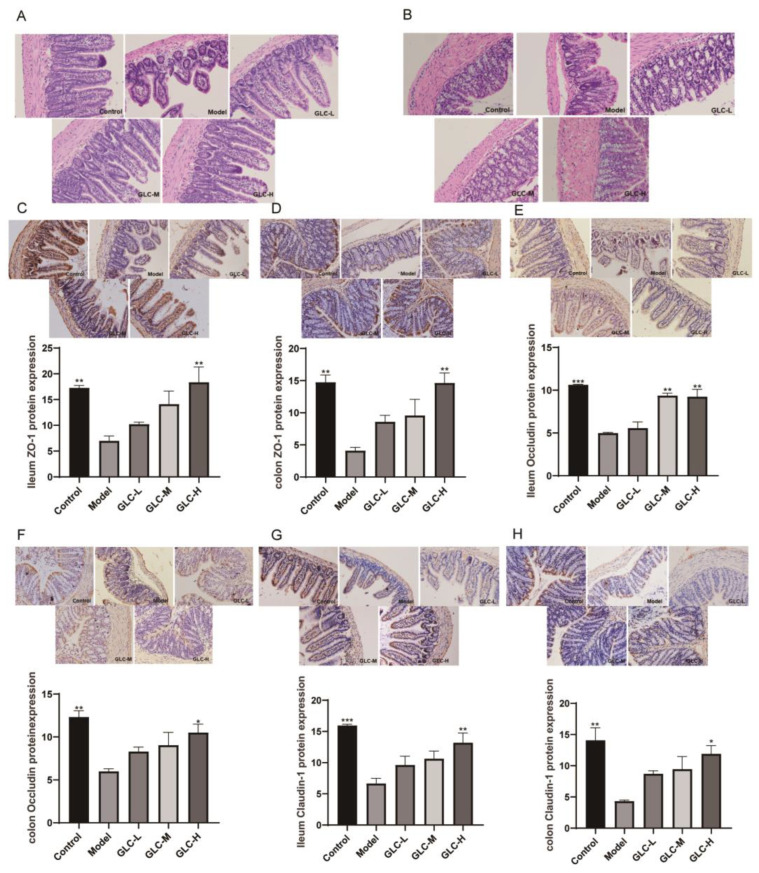
The effect of GLC on intestinal barrier function. (**A**) H&E staining of sections of ileum (200×) and (**B**) colon (200×). (**C**) The protein expression levels of ileum ZO-1, (**D**) colon ZO-1, (**E**) ileum Occludin, (**F**) colon Occludin, (**G**) ileum Claudin-1, and (**H**) colon Claudin-1. The data are presented as the mean ± SEM (n = 3). * *p* < 0.05, ** *p* < 0.01, *** *p* < 0.001 compared to the model group.

The effect of GLC administration on the intestinal barrier structure in NAFLD mice was evaluated via pathological analysis by H&E staining of mouse intestinal sections (Figure 7A,B). The results showed the following: the ileum of the NAFLD mice showed impaired villus shedding, mucosal, and submucosal swelling and congestion; a reduced number of goblet cells; colonic crypt damage, specifically disruption of normal vertical crypt cell alignment; inflammatory infiltration of the lamina propria. GLC treatment ameliorated ileal villus structural disruption and colonic crypt malformations and improved intestinal integrity.

The downregulation of the tight junction proteins ZO-1, Occludin, and Claudin-1 can lead to the destruction of tight junction structures and an increase in cell bypass permeability, leading to LPS translocation to the liver and blood circulatory system and liver inflammation and injury [26]. The expression of Claudin-1 in the colon and ileum of NAFLD mice was detected and analyzed by IHC (Figure 7C–H). The results showed that the expression levels of ZO-1, Occludin, and Claudin-1 in the colon and ileum of the model mice were clearly decreased, while the protein expression levels of ZO-1, Occludin, and Claudin-1 in the colon and ileum of the mice were increased after GLC administration. These findings indicate that GLC administration effectively ameliorates the loss of integrity of the intestinal mucosal barrier in HFHSD-fed NAFLD mice by increasing the expression of Claudin-1.

## 3. Discussion

We observed the morphology of mouse liver tissue and found that the lipid spots on the surface of the mouse livers improved after GLC administration. The H&E staining and oil red O staining results showed that GLC administration reduced lipid accumulation in the liver and alleviated liver steatosis. Whether in the theoretical context of “multiple strikes” or “secondary strikes”, the accumulation of toxic lipids in the liver is an important factor in the occurrence and development of NAFLD [27]. Therefore, we believe that GLC can play a specific role in treating lipid metabolism in NAFLD mice. Lipid metabolism in the liver is regulated by multiple factors. The imbalance of one or more of these factors may disrupt the lipid metabolism process, leading to lipid accumulation [7,28]. We measured the TC, TG, LDL-C, and HDL-C levels in serum and liver, and the results showed that GLC can reduce lipid metabolism in NAFLD mice. Hepatocytes are the main type of parenchymal cells in the liver, and hepatic steatosis is the result of an imbalance in lipid metabolism in hepatocytes. Therefore, abnormal lipid metabolism can affect the occurrence and development of NAFLD. Our results preliminarily explore the therapeutic effect of GLC on NAFLD through its regulatory effect on lipid metabolism. Fatty acids in the blood or synthesized by DNL of liver lipids are consumed [8]. The fatty acids in the liver are mainly derived from FFA produced via TG decomposition of adipose tissue TG, which enter the liver through the blood circulatory system and the levels of which are mainly regulated by insulin. IR caused by damage to the insulin receptor and other factors leads to the mobilization of FFAs from adipocytes with excessive fat to the liver [29], which leads to the accumulation of fatty acids in the liver and ultimately promotes the development of NAFLD [8,30]. Our results showed that after GLC administration, the TG level in serum and liver decreased, and the FFA levels in serum decreased. Therefore, GLC administration exerts a certain regulatory effect on the accumulation of fatty acids in NAFLD mice. IR can lead to hyperinsulinemia, which is mediated by insulin to inhibit liver glucose production imbalance, leading to an increase in blood sugar levels, which also helps to provide a substrate for DNL [31]. In the case of insulin resistance, the inhibition of fat breakdown by insulin can be hindered, leading to an increase in circulating FFA levels. Therefore, abnormal lipid metabolism can affect NAFLD [32]. After GLC treatment, the symptoms of hyperinsulinemia and hyperglycemia in mice were reduced. Further PCR experiments revealed that GLC reduced the expression of SREBP-1c, PPARγ, and ACC, while increasing the expression of CPT1. Our results show that GLC administration significantly improves the abnormal lipid metabolism and IR of NAFLD mice and alleviates the pathological conditions of liver lipid accumulation and steatosis in mice, indicating that GLC exerts a certain improvement effect on NAFLD.

The significant increase in AST and ALT levels in NAFLD mice indicates that liver disease has progressed from a state with simple lipid accumulation to a state of injury [33]. GLC administration significantly reduced the levels of AST and ALT in NAFLD mice, indicating that GLC can, to some extent, reverse the liver injury and improve liver function in NAFLD mice. Inflammation can cause a stress response in liver cells, induce lipid accumulation, and is an important pathological factor in the occurrence and development of NAFLD [12,34]. This study evaluated the effect of GLC administration on the systemic inflammatory response in NAFLD mice by measuring serum inflammatory factor levels. The results showed that GLC treatment reduced proinflammatory factor levels, promoted anti-inflammatory factor release, and effectively alleviated the systemic inflammatory response in NAFLD mice. NAFLD is associated with the activation of many inflammatory pathways, and the mediators of the process may be derived from multiple sources. Inflammatory factors such as TNF-α, IL-1β, and IL-6 are important mediators involved in inflammation that can activate the inflammatory cascade, promote the abundant expression of proinflammatory factors, accelerate the occurrence and development of inflammatory reactions, and lead to secondary liver injury in the NAFLD context. A quantitative analysis of the relative mRNA expression levels of the liver inflammatory factor IL-10, IL-6, TNF-α, and IL-1β indicated that GLC administration may slow liver inflammation by regulating the balance between proinflammatory (IL-6, TNF-α, and IL-1β) and anti-inflammatory factor (IL-10) expression.

Activation of the NF-κB pathway is crucial to the inflammatory response in the NAFLD liver and can regulate the expression of various inflammation-related factors [35,36]. NF-κB can be regulated by various inflammatory factors, such as TNF-α and IL-1β. After activation, NF-κB can enhance the expression of these cytokines, and excessive expression of these cytokines further promotes the activation of NF-κB, ultimately forming a vicious cycle leading to a continuous inflammatory response. Our results show that after GLC administration, serum IL-6 and TNF-β levels decreased, while the IL-10 level increased. Further WB experiments showed that after GLC administration, the expression of NF-κB and p-NF-κB decreased. The TLR4 protein is a transmembrane protein in the cell membrane. It activates Kupffer cells mainly through the MYD88-dependent pathway, thereby activating the NF-κB pathway and finally activating the inflammatory cascade [37]. In addition, TLR4 is an important downstream membrane CD14 partner. In this study, we quantitatively analyzed the expression of liver NF-κB, p-NF-κB, TLR4, MYD88, and CD14 protein levels through WB assays. It was found that GLC administration reduced the expression of various proteins. The results indicate that GLC administration may attenuate the inflammatory process in the livers of HFHSD-fed NAFLD mice by regulating the TLR4/NF-κB pathway and regulating the expression of related inflammatory factors in the pathway.

The intestine is considered the first barrier against bacteria, while the liver is the second barrier because the liver and intestine are anatomically and functionally similar [38,39]. Through a pathological analysis performed with H&E staining of the intestinal tissue of mice, we found that the intestinal integrity of the NAFLD mice was disrupted, and GLC administration increased intestinal integrity. The portal vein system is located at the interface between the host and inflammatory mediators present in the intestine, and intestine-derived LPS can enter the liver through the portal vein [40]. Under normal physiological conditions, the intestine is the largest reservoir of intestine-derived endotoxins in the human body. Excessive growth of small intestinal bacteria or increased intestinal permeability can lead to the translocation of bacteria and their byproducts (including LPS) and activate Kupffer cells and hepatic stellate cells through the LPS/TLR4 pathway, leading to inflammatory reactions [41]. In this study, we measured LPS in serum, and the results showed that GLC administration effectively improves LPS translocation to the blood circulatory system via the intestine of NAFLD mice. Further analysis of the changes in intestinal permeability and inflammation in NAFLD mice was performed through RT-PCR and immunohistochemistry. This study measured LPS in serum, and the results showed that GLC administration can effectively improve LPS translocation to the blood circulation in the intestine of NAFLD mice. Intestinal barrier function was analyzed, and the results indicate that GLC administration effectively increased the integrity of the intestinal mucosal barrier in HFHSD-fed NAFLD mice by upregulating the expression of the tight junction proteins ZO-1, Occludin, and Claudin-1. Therefore, GLC administration enhances intestinal barrier function, reduces LPS translocation to the blood circulatory system, and ultimately improves liver function in NAFLD mice. In recent years, safe and reliable drugs have attracted widespread interest [42]. In summary, GLC, as a natural sugar, has a high-safety profile and induces few toxic side effects. GLC has been extensively studied in the treatment of inflammation. We investigated the effect of GLC on NAFLD from the perspective of its effect on lipid metabolism, the inflammatory response, and intestinal barrier function, and found that GLC has a therapeutic effect on NAFLD. Therefore, GLC is expected to become an effective drug for treating NAFLD.

## 4. Materials and Methods

### 4.1. Materials

An HFHSD (23.3% protein, 46% carbohydrate, and 20.4% fat) was purchased from Research Diets Company, D12327 (New Brunswick, NJ, USA).

Glucosamine (GLC; D-glucosamine hydrochloride, biotech grade, CAS number: 66-84-2) was purchased from Shanghai McLean Biochemical Technology Co., Ltd. (Shanghai, China).

Metformin was purchased from Sino-US Shanghai Squibb Pharmaceutical Co., Ltd. (CAS number: ABH5474) (Shanghai, China).

The primary antibodies used were an anti-TLR4 monoclonal antibody (66350-1-Ig, 1:1000), anti-CD14 monoclonal antibody (60253-1-Ig, 1:1000), anti-MYD88 monoclonal antibody (67969-1-Ig, 1:1000), anti-GAPDH polyclonal antibody (10494-1-AP, 1:5000), anti-ZO-1 polyclonal antibody (21773-1-AP, 1:100), anti-Occludin polyclonal antibody (27260-1-AP, 1:100), and anti-Claudin-1 polyclonal antibody (13050-1-AP, 1:500). The above antibodies were purchased from Proteintech (Wuhan, China). An anti-NF-κB p65 polyclonal antibody (ab16502, 1:1000) and anti-NF-κB p65 (phosphor-S536) polyclonal antibody (ab76302, 1:1000) were purchased from Abcam Company (Cambridge, UK).

### 4.2. Animals and Experimental Design

The experimental animals were C57BL/6 male mice (SPF grade, 7 weeks old) provided by Hunan Slack Jingda Laboratory Animal Co., Ltd. (Hunan, China) (animal production license number: SCXK (Guangdong) 2019-0004). The mice were raised in the SPF laboratory of the Experimental Animal Center of Guangdong Pharmaceutical University (license number: SYXK (Guangdong) 2017-0125).

The animal research protocol was approved by the Ethics Committee of the Experimental Animal Center of Guangdong Pharmaceutical University. The animal ethical review number is GDPULACSOF2017378.

Sixty 7-week-old C57BL/6 male mice were adaptively reared for 1 week. Initially, they were randomly divided into a blank group (n = 10) and a model group (n = 50). The control group (Control) was fed a normal chow diet, and the model group was fed a HFHSD. After rearing for 8 weeks, 6 mice were randomly selected from each of the two groups, blood was collected from the tails of these mice, and the relevant indicators were measured. After the successful modeling of NAFLD in the mice, the mice (n = 50) were randomly divided into 5 groups with 10 mice in each group: the model group (Model), the positive control drug metformin group (metformin group; 50 mg/kg/d metformin), the high-dose glucosamine group (GLC-H; 600 mg/kg/d), the medium-dose glucosamine group (GLC-M; 300 mg/kg/d), and the low-dose glucosamine group (GLC-L; 150 mg/kg/d). Each dose group was given a corresponding dose of drug based on the weight of the mice per day, while the other groups of mice were given an equal amount of ultrapure water. The whole process of modeling and GLC administration was carried out in an SPF laboratory with alternating light and dark conditions within a 24 h period. The temperature was 22–26 °C, and the humidity was 55 ± 5%. During the animal experiments, the mice were weighed, and their weights were recorded at regular intervals every week. The uneaten feed and the weight of the feed of each group of mice were recorded at regular intervals every week, and the total and average feed intake levels of each group of mice were calculated. The mice were anesthetized after 12 weeks by gavage, and blood was collected through eye blood sampling. Then, the mice were euthanized using the cervical dislocation method. Mouse livers, cecal contents, feces, and other samples were collected and stored at −80 °C for future use.

### 4.3. Serum Biochemical Analyses

For serum index measurement, blood samples were maintained at room temperature for 30 min and centrifuged at 4 °C and 3000 rpm for 15 min to collect the upper fraction (serum). The TC, TG, HDL, LDL, FFA, GLU, AST, ALT, CAT levels and the T-AOC were measured according to the steps in the assay kit instructions from Nanjing Jiancheng.

### 4.4. Hepatic Biochemical Assays

We precisely weighed 0.1 g of liver tissue with a 1/1000 analytical balance, added 0.9 mL of normal saline to the sample, chopped the tissue into pieces, homogenized the tissue with an automatic homogenizer, and centrifuged the sample at 2500 rpm for 10 min. The supernatant was collected, and biochemistry kits were used to determine the liver lipid metabolism-related indexes TC, TG, HDL, and LDL and the antioxidant function-related indexes GSH, CAT, SOD, and MDA of the mice (Nanjing Jiancheng Biomedical Company, Nanjing, China).

### 4.5. ELISA

Blood samples were maintained at room temperature for 30 min and centrifuged at 4 °C and 3000 rpm for 15 min. The upper fraction (serum) was collected. Measurement of systemic inflammatory factors (IL-6, IL-10, and TNF-α), insulin, and LPS levels was performed using ELISA kits (Shanghai Enzyme-linked Biotechnology Co., Ltd.) (Shanghai, China).

### 4.6. Histological Staining

Fresh liver, ileum, and colon tissues were placed in a 4% paraformaldehyde solution for fixation. Then, the samples were embedded in high melting point paraffin and sliced at a thickness of 4 mm. Finally, the sections were stained with hematoxylin hematoxylin and eosin (H&E). The liver tissue was placed in an optimal cutting temperature compound (Sakura, Torrance, CA, USA) for embedding, and then cut into 10 um thick sections for oil red O staining. All samples were photographed under an Olympus BX51 system.

### 4.7. Quantitative Real-Time PCR

Total RNA was extracted from liver tissue according to the instructions from the manufacturer of TRIzol reagent (TaKaRa, Dalian, China). cDNA was transformed from 1 ug of total RNA using a reverse transcription kit (TaKaRa, Dalian, China). The reverse-transcribed cDNA and corresponding gene primers were obtained following the instructions of a TaKaRa TB Green Premix Ex TaqTM II Kit (TaKaRa, Dalian, China), and then analyzed in a LightCycle 480 machine.

The expression level data were normalized to the expression level of GAPDH, and the 2-ΔΔ Ct method was used for calculating the expression. The primer sequences are listed in Table 1.

### 4.8. Western Blotting

Cold RIPA buffer (Meilunbio^®^, Dalian, China) was added to the liver tissue. Then, the samples were centrifuged at 12,000 rpm for 10 min at 4 °C. The protein concentration of the sample was measured according to the instructions of a BCA assay kit (Beyotime Biotechnology, Shanghai, China). Next, SDS-PAGE was used to separate the proteins, which were transferred into PVDF membranes. The PVDF membranes were blocked with 5% skim milk. The corresponding primary antibody was added to the membranes and incubated overnight in a refrigerator at 4 °C. After incubation with the primary antibody, the secondary antibodies were incubated at room temperature for 90 min. Enhanced chemiluminescence (ECL) reagent was added to the incubated membranes for imaging, and ImageJ software (ImageJ version 1.48 (National Institutes of Health, Bethesda, MD, USA)) was used for data analysis.

### 4.9. Immunohistochemistry

The wax was removed from the ileum and colon tissue sections, which were subjected to antigen repair. Then, the tissue slices were blocked with serum. A primary antibody was added to the tissue, and the slides with tissue were placed in a cassette and then incubated overnight in a 4 °C refrigerator. Then, secondary antibodies were added to the tissue and incubated at room temperature for 1 h. After incubation, DAB chromogenic solution was added dropwise to the tissue for staining. All samples were photographed with an Olympus BX51 system. Image-Pro Plus 6.0 software (Media Cybernetics, Inc., Rockville, MD, USA) was used for data analysis.

### 4.10. Statistical Analysis

All experimental data were analyzed using GraphPad Prism 8.0 (San Diego, CA, USA), and expressed as the mean ± SEM. Statistical analysis was performed using one-way ANOVA followed by Duncan’s multiple-comparison test. Differences with *p* < 0.05 were considered statistically significant.

## 5. Conclusions

In conclusion, GLC attenuates liver lipid accumulation, alleviates IR, increases liver function, and alleviates systemic oxidative stress and the inflammation response in NAFLD mice. The mechanism may be triggered by improving intestinal barrier function, regulating the LPS/TLR4/NF-κB inflammatory pathway, and improving liver lipid metabolism.

## Figures and Tables

**Table 1:** Primer sequences.

Gene	Forward Primer	Reverse Primer
ACC	GGCCAGTGCTATGCTGAGAT	AGGGTCAAGTGCTGCTCCA
CPT1	AGGACCCTGAGGCATCTATT	ATGACCTCCTGGCATTCTCC
SREBP1c	GGAGCCATGGATTGCACATT	GGCCCGGGAAGTCACTGT
PPARγ	TCTCCATGACAGACATGGACA	GTCAGGCTGTTGGTCTCACA
GADPH	GGGAAGCCCATCACCATCTTC	AGAGGGGCCATCCACAGTCT
IL-1β	TGGACTTCGCAGCACAAAATG	CACTTCACGCTCTTGGATGA
IL-6	AGACCGCTGCCTGTCTAAAA	TTTGATGTCGTTCACCAGGA
TNF-α	CCCACACCGTCAGCCGATTT	GTCTAAGTACTTGGGCAGATTGACC

## Data Availability

The data from the present study are available in the article.

## Sample Availability

Samples of the compounds are available from the authors.
